# Kyphosis and paraspinal muscle composition in older men: a cross-sectional study for the osteoporotic fractures in men (MrOS) research group

**DOI:** 10.1186/1471-2474-15-19

**Published:** 2014-01-16

**Authors:** Wendy B Katzman, Dana Miller-Martinez, Lynn M Marshall, Nancy E Lane, Deborah M Kado

**Affiliations:** 1 Department of Physical Therapy and Rehabilitation Science, University of California, San Francisco, CA 94158, USA; 2 UCLA David Geffen School of Medicine, Division of Geriatrics, University of California, Los Angeles, USA; 3 Oregon Health and Science University, School of Medicine, Orthopaedics & Rehabilitation Department, Portland, OR, USA; 4 Department of Internal Medicine, University of California, Davis, CA, USA; 5 Departments of Family and Preventive Medicine and Internal Medicine, University of California, San Diego, CA, USA

**Keywords:** Kyphosis, Hyperkyphosis, Spinal muscle composition, Vertebral fractures, BMI

## Abstract

**Background:**

The prevalence of hyperkyphosis is increased in older men; however, risk factors other than age and vertebral fractures are not well established. We previously reported that poor paraspinal muscle composition contributes to more severe kyphosis in a cohort of both older men and women.

**Methods:**

To specifically evaluate this association in older men, we conducted a cross-sectional study to evaluate the association of paraspinal muscle composition and degree of thoracic kyphosis in an analytic cohort of 475 randomly selected participants from the Osteoporotic Fractures in Men (MrOS) study with baseline abdominal quantitative computed tomography (QCT) scans and plain thoracic radiographs. Baseline abdominal QCT scans were used to obtain abdominal body composition measurements of paraspinal muscle and adipose tissue distribution. Supine lateral spine radiographs were used to measure Cobb angle of kyphosis. We examined the linear association of muscle volume, fat volume and kyphosis using loess plots. Multivariate linear models were used to investigate the association between muscle and kyphosis using total muscle volume, as well as individual components of the total muscle volume, including adipose and muscle compartments alone, controlling for age, height, vertebral fractures, and total hip bone mineral density (BMD). We examined these associations among those with no prevalent vertebral fracture and those with BMI < 30 kg/m^2^.

**Results:**

Among men in the analytic cohort, means (SD) were 74 (SD = 5.9) years for age, and 37.5 (SD = 11.9) degrees for Cobb angle of kyphosis. Men in the lowest tertile of total paraspinal muscle volume had greater mean Cobb angle than men in the highest tertile, although test of linear trend across tertiles did not reach statistical significance. Neither lower paraspinal skeletal muscle volume (p-trend = 0.08), or IMAT (p-trend = 0.96) was associated with greater kyphosis. Results were similar among those with no prevalent vertebral fractures. However, among men with BMI < 30 kg/m^2^, those in the lowest tertile of paraspinal muscle volume had greater adjusted mean kyphosis (40.0, 95% CI: 37.8 – 42.1) compared to the highest tertile (36.3, 95% CI: 34.2 – 38.4).

**Conclusions:**

These results suggest that differences in body composition may potentially influence kyphosis.

## Background

Hyperkyphosis, an exaggerated curvature in the thoracic spine, is commonly observed in the older population although its causes have not been well investigated. Greater degrees of kyphosis impairs physical function [1-5] and well-being [6], and increases load on the vertebral bodies [7,8] that potentially could increase the risk for vertebral compression fractures. A recent study confirms the need for more comprehensive assessment of health outcomes in older adults with greater degree of kyphosis [9]. Hyperkyphosis is considered a problem that primarily affects older women, even though the prevalence of hyperkyphosis in older men is estimated to range from 15-40%, depending upon how kyphosis is defined [6,10]. While commonly recognized risk factors for hyperkyphosis in women include advanced age, low bone mass and underlying vertebral fractures [11], only 1/3 of older women with the most severe kyphosis have vertebral compression fractures (VCFs) [12], suggesting other factors contribute to kyphosis that have not been well defined. Furthermore, it is not known whether men have similar risk factors for hyperkyphosis.

Recently Katzman, et al. determined that paraspinal trunk muscle composition is associated with hyperkyphosis in a cohort of older community-dwelling men and women [10]. Among individuals with a greater degree of kyphosis, there was more adipose accumulation within the paraspinal muscles. Imaging methods that include computed tomography and MRI are the most accurate methods available for quantifying skeletal muscle composition in vivo, and these methods permit measurement of skeletal muscle and adipose tissues within the muscle fascia [13]. While MRI does not expose an individual to radiation, it overestimates the amount of adipose tissue within the erector spinae muscle fascia [14], suggesting that CT imaging of the erector spinae may be more precise than MRI. Attenuation refers to the energy of the x-ray beam as it passes through the tissue, and whereas all tissue attenuates the x-ray beam, muscle attenuates more than adipose. Attenuation indicates the density of muscle with lower attenuation indicative of greater lipid infiltration [15]. An attenuation value, called a Hounsfield Unit (HU), is assigned to each volume element (or voxel) in the CT image. Previous work demonstrated that muscle and adipose tissues have distinct HU ranges [15-18]. Thus, CT imaging permits segmentation of the total muscle volume contained within the fascial borders into its skeletal muscle and adipose tissue components.

Skeletal muscle composition changes with aging and differs according to sex. During aging, adipose tissue volume increases, skeletal muscle tissue volume declines, and the remaining muscle tissue is susceptible to lipid infiltration [19]. Younger adults have greater muscle volume and higher muscle attenuation than older adults [20]. Moreover, sex also affects muscle composition of the skeletal muscles, particularly in the paraspinal muscles, and men have higher paraspinal muscle attenuation than women [21,22]. Given these age-related changes in paraspinal muscle composition and the differences in paraspinal attenuation in men compared with women, further investigation of the association of paraspinal muscle composition with kyphosis in older men is warranted.

We conducted a cross-sectional study to investigate differences in degree of thoracic kyphosis according to paraspinal muscle composition in older men using data from the Osteoporotic Fractures in Men (MrOS) Study. We hypothesized that both lower paraspinal muscle volume and higher paraspinal intermuscular adipose volume would be associated with greater degree of Cobb angle of kyphosis, independent of known and potential covariates age, weight, height, abdominal fat, hip bone mineral density, and prevalent vertebral fractures. We further hypothesized that obese men would have more paraspinal muscle volume than men with BMI < 30 kg/m^2^ that would have a protective effect on Cobb angle of kyphosis, thus warranting examining these associations according to BMI.

## Methods

### Participants

The Osteoporotic Fractures in Men (MrOS) cohort consists of 5,994 men aged 65 or older who were recruited from 6 academic medical centers in Birmingham, AL; Minneapolis, MN; Palo Alto, CA; Pittsburgh, PA; Portland, OR; and San Diego, CA [23,24]. The primary purpose of MrOS has been to describe the risk factors for osteoporotic fractures in older men. Funding for MrOS began in July 1999 and the study is now its 13th year. At baseline, all men were referred for plain thoracic and lumbar radiographs and 3786 men were referred for quantitative computed tomography (QCT) scans of the hips, lumbar spine and abdomen. Abdominal body composition measures were performed for a random sample of 667 participants in the sample of 3786 who had been referred for QCT scans at baseline [25]. The analytic cohort for our study included the 475 randomly selected participants from the MrOS study with both baseline abdominal body composition measures from QCT scans and plain thoracic radiographs.

Approval of the conduct of the MrOS study was obtained from the Institutional Review Board of each of the participating medical centers and written informed consent was obtained from all study participants.

### Muscle variables

**Figure 1:**
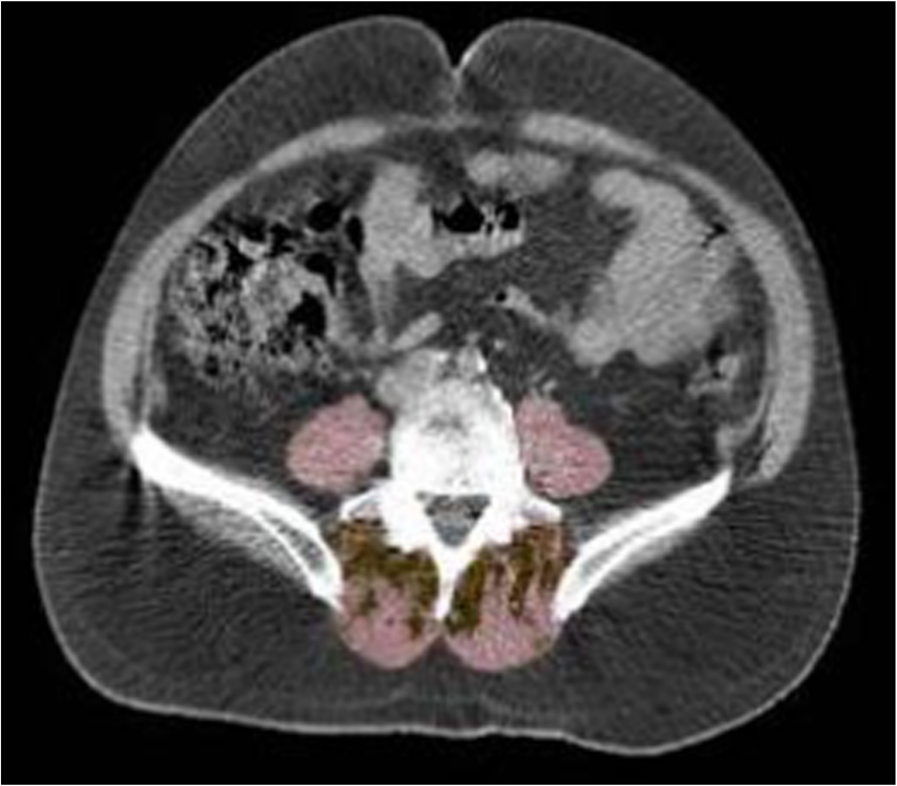
**Paraspinal muscle group including the longissimus, iliocostalis and multifidus muscles (posterior to the vertebral body) and psoas muscle (lateral to the vertebral body) with intermuscular adipose tissue (IMAT) shown at L4/L5 interspace by abdominal QCT.** Pink = muscle tissue; yellow = IMAT.

Baseline abdominal QCT scans were used to obtain abdominal body composition measurements of abdominal wall muscle and adipose tissue distribution. Abdominal scans were obtained using a standardized protocol which specified scanning from the mid-L3 to the mid-L5 vertebra at 5-mm slice thickness with participants in the supine position. We completed abdominal body composition measurements on the scan at the L4-L5 intervertebral disc level. Scans were processed by two specialists at Oregon Health and Science University (OHSU) using a standardized protocol as previously described [26]. Briefly, a spline tool was used to trace the outer and inner fascial borders of the abdominal wall musculature comprising 6 bilateral muscle groups - the rectus abdominis, external oblique, internal oblique, transverse abdominis, psoas and paraspinals. The paraspinal muscle group included the muscles on each side of the vertebral body in the lumbar region located lateral to the lumbar vertebral spinous process and posterior to the lumbar vertebral transverse processes comprising the longissimus, iliocostalis and multifidus muscles. Intermuscular adipose tissue (IMAT) included adipose tissue located around muscle bundles within each muscle group (Figure 1). Attenuation ranges used to estimate volumes (cm^3^) of muscle were 0 to 100 HU [15,18] and of IMAT were -190 to -30 HU [16,17]. Thus, for all muscle groups studied, we had three volume variables: skeletal muscle only, IMAT, and total volume which is the sum of skeletal muscle plus IMAT volume. Muscle attenuation (HU) was obtained for the total volume and the skeletal muscle only volume. To account for variability in image attenuation across scanners, the HU in the scans were scaled to the calibration standard for water density (0 mg/cm^3^). Inter- and intra-rater reliability was monitored throughout image processing with intraclass correlation coefficients (ICC). Final intra-rater ICCs for all measures were ≥0.98 for rater 1 and ≥0.95 for rater 2, and final inter-rater ICCs were all ≥0.96.

### Cobb angle of kyphosis measurements

**Figure 2:**
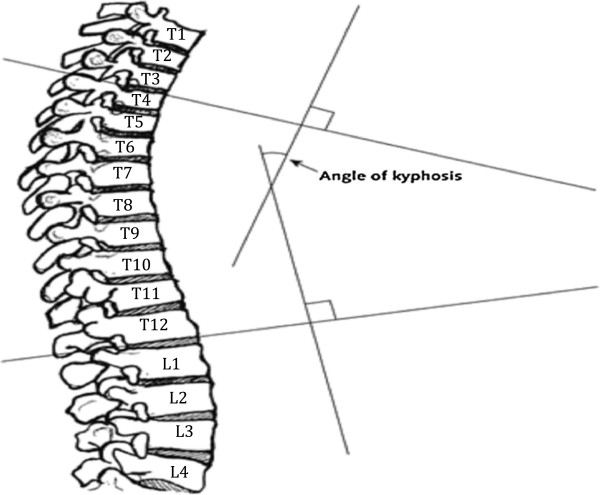
Cobb angle of kyphosis, measured as the angle of intersecting lines drawn from the superior endplate of T4 and the inferior endplate of T12, calculated from a radiographic image shown.

The Cobb angle of kyphosis was measured from supine lateral spine radiographs taken during baseline visit 1. We used the modified Cobb method [27] with a fixed cut-off of T4 and T12, largely because T1 to T3 are usually not well visualized on lateral spine films due to the overlying shoulders and scapulae interfering with the projection. Technicians placed 6 points corresponding to the 4 corners of the vertebral body and the midpoints of the endplates on each vertebral body from T4 to T12. From the superior surface of T4 and inferior surface of T12, a computerized digitization program erected perpendicular lines whose intersection was the kyphotic angle (Figure 2). If for any reason T4 or T12 were not visible, the next adjacent visible vertebra (T5 if T4 not visible or T11 if T12 not visible) was used as an alternative. The ICC for Cobb angle of kyphosis has been previously reported 0.99 [27].

### Vertebral fracture measurements

Vertebral fractures were adjudicated from baseline lateral lumbar and thoracic spine radiographs based upon a previously developed protocol [28]. The SpineAnalyzer™ (Optasia Medical Ltd., Cheadle, UK) workflow tool was used to automate placement of 6-point morphometric points on each radiograph in order to efficiently identify the vertebral bodies for evaluation. An expert radiologist then graded each vertebra as normal (0), mild (1), moderate (2), severe (3) using the well-established, highly reliable semi-quantitative Genant method [29]. Prevalent vertebral fractures were defined as grade 2 or 3.

### Anthropometric measurements

Body weight and height were taken at baseline by an examiner using standard equipment, including a Harpenden stadiometer and balance beam scale while wearing indoor street clothing without shoes [23]. Body mass index (BMI) was calculated as weight divided by the square of height in meters (kg/m^2^). Bone mineral density (BMD) was also measured at baseline in the proximal femur and lumbar spine using DXA measured by Hologic QDR 4500 densitometers.

### Statistical analysis

We built models to control for potential confounding using an empirical approach. We examined the association between an *a priori* group of potential confounders and Cobb angle and based on the change in the regression coefficient, we used p value <0.1 to select confounders including age, height, vertebral fractures, and total hip bone mineral density (BMD). Loess plots were used initially to determine the shape of the relations of muscle volume, adipose volume and Cobb angle. Because associations were not linear, muscle mass measurements were divided into quintiles, quartiles and tertiles to investigate non-linear trends in kyphosis, but due to the limited sample size, we used tertiles for our analysis. Multivariate linear models were used to investigate the association between the muscle variables and kyphosis using total muscle volume, as well as individual components of the total muscle volume that included the IMAT and skeletal muscle compartments alone. All multivariable analyses controlled for age, height, vertebral fractures, and total hip BMD. These associations were also investigated separately for the total abdominal (rectus abdominis, transversus abdominis, internal obliques and external obliques), psoas and paraspinal muscle groups. We examined these associations among those with no prevalent vertebral fracture (n = 429) and among those with BMI < 30 kg/m^2^ (n = 394). We conducted sensitivity analyses with different adjustment variables as well as an alternate definition of hyperkyphosis >40 degrees.

## Results

The subject characteristics of the overall MrOS cohort, means (standard deviation), were 74 (SD = 5.9) years for age, 174 (SD = 6.8) cms for height, 0.96 (SD = 0.14) g/cm^2^ for total hip BMD, 27 (SD = 3.8) kg/m^2^ for BMI, and 7.5% had prevalent vertebral fractures. In the analytic cohort of 475 men, means (SD) were 74 (SD = 5.9) years for age and 37.5 (SD = 11.9) degrees for Cobb angle of kyphosis (Table 1). Men with BMI < 30 kg/m^2^ were older, had greater Cobb angle, lower hip BMD, and lower total abdominal muscle volume, but no difference in total paraspinal or total psoas muscle volume. When we separated total muscle volume into its skeletal muscle tissue and IMAT components, skeletal muscle volume but not attenuation in all of the abdominal wall muscle groups was greater, and IMAT in the abdominal and psoas muscle groups was lower in men with BMI < 30 kg/m^2^.

**Table 1:** Baseline characteristics of MrOs sample (n = 475)

	**Full sample mean (SD)**	**Stratified by BMI mean (SD)**		
		**BMI < 30 (n = 399)**	**BMI ≥30 (n = 76)**	**p-value for difference**^**a**^
Age (years)	74.2 (5.86)	74.7 (5.86)	71.6 (5.09)	<.001
Cobb angle of kyphosis (degrees)	37.5 (11.90)	37.9 (12.0)	35.2 (11.3)	.07
Height (cm)	173.6 (6.97)	173.7 (7.19)	172.9 (5.72)	.28
Total hip BMD (g/cm^2^)	0.94 (0.14)	0.92 (0.14)	1.01 (0.13)	<.001
BMI (kg/m^2^)	26.8 (3.21)	25.8 (2.37)	32.0 (1.54)	<.001
Prevalent vertebral fracture (% yes)	9.68	10.5	5.26	0.16
Total paraspinal muscle volume (cm^3^)	18.6 (3.79)	18.7 (3.69)	18.0 (4.28)	0.15
Paraspinal muscle volume (cm^3^)	12.1 (3.47)	12.3 (3.42)	11.4 (3.64)	0.04
Paraspinal muscle attenuation (HU)	43.6 (5.48)	43.5 (5.40)	43.9 (5.91)	0.57
Paraspinal adipose tissue volume (cm^3^)	6.41 (2.61)	6.36 (2.65)	6.62 (2.37)	0.43
Total psoas muscle volume (cm^3^)	11.1 (2.65)	11.1 (2.58)	10.7 (2.98)	0.15
Psoas muscle volume (cm^3^)	10.0 (3.16)	10.2 (2.99)	8.82 (3.76)	.003
Psoas muscle attenuation (HU)	51.53 (5.13)	51.64 (5.43)	50.98 (3.05)	0.14
Psoas adipose tissue volume (cm^3^)	1.07 (0.91)	0.92 (0.77)	1.85 (1.15)	< .001
Total abdominal muscle volume (cm^3^)	63.9 (8.11)	63.5 (7.82)	65.8 (9.29)	.048
Abdominal muscle volume (cm^3^)	48.3 (10.3)	48.9 (9.82)	45.2 (12.0)	.01
Abdominal muscle attenuation (HU)	45.79 (4.36)	45.84 (4.54)	45.56 (3.30)	0.53
Abdominal adipose tissue volume (cm^3^)	15.6 (6.01)	14.6 (5.50)	20.6 (6.14)	< .001

^a^p-value represents *t*-test (for continuous variables) or Chi-square test (for categorical variables) for difference between those with BMI < 30 kg/m^2^ versus those with BMI ≥ 30 kg/m^2^.

cm = centimeters; cm^3^ = centimeters^3^; g/cm^2^ = grams/centimeter^2^; kg/m^2^ = kilograms/meter^2^; HU = Hounsfield units.

Men in the lowest tertile of total paraspinal muscle volume had greater mean Cobb angle than men in the highest tertile (Table 2), although the test of linear trend across tertiles did not reach statistical significance. When we further separated total paraspinal volume into its skeletal muscle tissue and IMAT components, neither lower paraspinal skeletal muscle volume (p-trend = 0.08), or IMAT (p-trend = 0.96) was associated with greater kyphosis. Results were similar among those with no prevalent vertebral fractures. However, among men with BMI < 30 kg/m^2^, those in the lowest tertile of paraspinal skeletal muscle volume had greater adjusted mean kyphosis (40.0, 95% CI: 37.8 – 42.1) compared to men in the highest tertile (36.3, 95% CI: 34.2 – 38.4). There were no associations of the total abdominal or total psoas muscle volumes with Cobb angle of kyphosis (Table 3), and no significant associations of the adjusted mean Cobb angle of kyphosis according to abdominal and psoas skeletal muscle and IMAT volumes, p > .05 (data not shown).

**Table 2:** Adjusted mean Cobb angle of kyphosis according to volumes of paraspinal muscle and intermuscular adipose tissue (IMAT) among 475 men aged ≥65 years

	**Least square mean (95% CI) for Cobb angle of kyphosis (degrees) within tertile of muscle measure**			
**Muscle volume tertile (from lowest to highest)**	**1**	**2**	**3**	**p-trend**
**Full analytic sample**^**a**^				
**MODEL 1**				
Total paraspinal muscle volume(includes paraspinal muscle and IMAT volume combined)	38.2 (36.4, 40.1)	38.2 (36.4, 40.0)	36.1 (34.2, 38.0)	0.12
**MODEL 2**				
Paraspinal Muscle Volume	38.8 (36.9 – 40.7)	37.3 (35.5 – 39.2)	36.3 (34.4 – 38.2)	0.08
Paraspinal IMAT Volume	37.6 (35.7 – 39.5)	37.4 (35.5 – 39.2)	37.5 (35.6 – 39.4)	0.96
**Among those with no prevalent vertebral fracture**^**b**^				
**MODEL 1**				
Total paraspinal muscle volume (includes paraspinal muscle and IMAT volume combined)	37.5 (35.6, 39.4)	37.8 (35.9, 39.6)	35.9 (33.9, 37.9)	0.28
**MODEL 2**				
Paraspinal Muscle Volume	38.5 (36.6 – 40.5)	36.6 (34.7 – 38.5)	36.1 (34.2 – 38.1)	0.10
Paraspinal IMAT Volume	36.9 (34.9 – 38.8)	37.3 (35.4– 39.1)	37.1 (35.1 – 39.1)	0.87
**Among those with BMI < 30 kg/m**^**2**^^**a**^				
**MODEL 1**				
Total paraspinal muscle volume (includes paraspinal muscle and IMAT volume combined)	39.0 (36.9, 41.2)	38.9 (36.9, 40.8)	35.9 (33.8, 38.0)	0.04
**MODEL 2**				
Paraspinal Muscle Volume	40.0 (37.8 – 42.1)	37.6 (35.7 - 39.6)	36.3 (34.2 – 38.4)	0.02
Paraspinal IMAT Volume	38.5 (36.3 - 40.6)	38.1 (36.1 - 40.1)	37.3 (35.2 - 39.5)	0.48

^a^Least square means and 95% CI estimated from multiple linear regression adjusted for age, height, prevalent vertebral fracture, and total hip BMD with paraspinal muscle volume and paraspinal IMAT in the same model. ^b^Least square means and 95% CI estimated from multiple linear regression adjusted for age, height, and total hip BMD with paraspinal muscle volume and paraspinal IMAT in the same model.

kg/m^2^ = kilograms/meter^2^.

**Table 3:** Adjusted mean Cobb angle of kyphosis according to volumes of abdominal muscle and psoas muscle among 475 men aged ≥65 years*

	**Least square mean (95% CI) for Cobb angle of kyphosis (degrees) within tertile of muscle measure**			
**Muscle volume tertile (from lowest to highest)**	**1**	**2**	**3**	**p-trend**
**Full analytic sample**^**a**^				
Total abdominal muscle volume	36.6 (34.7, 38.4)	38.8 (37.0, 40.7)	37.1 (35.2, 39.0)	0.69
Total psoas muscle volume	37.6 (35.8, 39.5)	38.0 (36.2, 39.8)	36.9 (35.0, 38.7)	0.56
**Among those with no prevalent vertebral fracture**^**b**^				
Total abdominal muscle volume	36.7 (34.7, 38.6)	38.0 (36.1, 39.8)	36.6 (34.6, 38.5)	0.94
Total psoas muscle volume	37.5 (35.6, 39.5)	37.5 (35.6, 39.3)	36.2 (34.3, 38.1)	0.32
**Among those with BMI < 30**^**a**^				
Total abdominal muscle volume	37.4 (35.3, 39.4)	39.0 (37.0, 40.9)	37.3 (35.1, 39.5)	0.97
Total psoas muscle volume	38.5 (36.4, 40.6)	37.9 (35.9, 39.9)	37.4 (35.4, 39.5)	0.48

*Total abdominal muscle volume and total psoas muscle volume are tested in separate models.

^a^Least square means and 95% CI estimated from multiple linear regression adjusted for age, height, prevalent vertebral fracture, and total hip BMD. ^b^Least square means and 95% CI estimated from multiple linear regression adjusted for age, height, and total hip BMD.

The sensitivity analysis where we replaced paraspinal IMAT with paraspinal muscle attenuation in models that also included paraspinal muscle volume showed similar findings. Specifically, neither paraspinal skeletal muscle volume or paraspinal muscle attenuation showed significant associations with Cobb angle, with the exception of paraspinal muscle volume among those with men with BMI < 30 (data not shown). Consistent with the results in Table 2, among men with BMI < 30 kg/m^2^, having less paraspinal muscle volume is associated with greater Cobb angle. In the multivariate adjusted model, when Cobb angle was dichotomized > 40 degrees, there was no association with paraspinal muscle volume or adipose volume, p > 0.05 (data not shown).

## Discussion

We found that paraspinal muscle volume influenced kyphosis among those with BMI < 30 kg/m^2^. Lower paraspinal muscle volume may reduce the capacity of paraspinal muscles to extend and stabilize the spine, resulting in increased kyphosis. In contrast, muscle volume in the abdominal and psoas muscles that predominantly flex the spine, did not influence kyphosis. Furthermore, we found that paraspinal muscle volume, rather than paraspinal muscle attenuation, a measure of intramuscular adipose tissue, had greater influence on kyphosis than IMAT in our cohort of older men.

These results are in contrast to our previous finding among older men and women in the Health Aging and Body Composition (Health ABC) Study that paraspinal muscle attenuation, not muscle volume, is associated with kyphosis. It is possible these differences could be because the Health ABC Study included men and women, and our MrOS cohort included men only who have greater muscle volume and attenuation than women. Our results could also be explained by differences in image acquisition and processing of the computed tomography scans that were used to obtain the muscle composition measurements; there are no standard methods, making comparisons of the Health ABC and MrOS findings difficult. Another hindrance to comparing results is that in our previously reported Health ABC study, muscle volume was categorized as the volume of non-bone, nonadipose tissue within the fascial plane of the specified muscle groups, whereas in our current MrOS study, muscle volume was defined as muscle and adipose tissue within the plane of the fascia inclusive of intramuscular adipose tissue. Furthermore, IMAT was not measured or included in the Health ABC analysis, whereas we controlled for IMAT in our analyses. However, when we did investigate paraspinal muscle attenuation in MrOS, it was not associated with Cobb angle of kyphosis. When we performed a sensitivity analysis and replaced IMAT with paraspinal muscle attenuation, the results did not appreciably change. In addition, while we used a continuous measure of Cobb angle of kyphosis in MrOS, in Health ABC we used a dichotomous variable of hyperkyphosis defined as >40 degrees, which could reflect a threshold effect of paraspinal muscle attenuation on Cobb angle of kyphosis. Nonetheless, we found no evidence of a threshold effect within the MrOS cohort. Finally, Health ABC measurements of Cobb angle were obtained from supine computed tomography scout scans, whereas in MrOS we obtained these measurements from supine lateral spine radiographs, and we do not know how well these measurements compare.

Vertebral fractures, commonly thought to be the cause of excessive kyphosis, did not influence the association between paraspinal muscle volume, paraspinal IMAT and Cobb angle of kyphosis. These results provide further evidence that factors other than vertebral fractures are responsible for greater degree of kyphosis. We found that paraspinal muscle volume, a factor that is potentially modifiable with exercise, is associated with Cobb angle of kyphosis in our cohort of older men with BMI <30 kg/m^2^ versus high BMI.

Whereas decreased paraspinal muscle attenuation, not muscle volume, has been associated with decline in physical performance among older adults [30,31], in our study, we found reduced paraspinal muscle volume, not muscle attenuation, influenced kyphosis. Resistance exercise trials targeting lower extremity muscle groups in older adults have reported that high-intensity resistance exercise produces large increase in muscle strength [32,33]. Improvement in lower extremity muscle strength following high intensity strengthening has also been associated with increased muscle volume and muscle attenuation [32-36]. Several randomized controlled trials of exercise have included back extensor muscle strengthening to reduce kyphosis [37-41], and it is possible that strengthening the back extensor muscles could improve paraspinal muscle volume and attenuation. Unfortunately the effects of back extensor muscle strengthening on paraspinal muscle volume or attenuation have not been investigated, and it is not known whether improving paraspinal muscle volume or attenuation reduces kyphosis.

While the phenotype of a smaller-framed older woman with hyperkyphosis is well-recognized, our findings suggest this phenotype also exists in older men. Body mass index, a proxy for human body fat based on an individual's weight and height, influenced kyphosis in our cohort of men. We found 83% of the men had BMI < 30 kg/m^2^, and among these men, low paraspinal muscle volume was associated with greater Cobb angle of kyphosis. In fact, among those with BMI < 30 kg/m^2^ and in the lowest tertile of paraspinal muscle volume, the mean kyphosis was 40.0 degrees (95% CI = 37.8-42.1), the threshold for defining hyperkyphosis in older adults [42,43]. Hyperkyphosis, low paraspinal muscle volume and BMI < 30 kg/m^2^ may reflect an underlying geriatric syndrome that impacts both older men and women. This kyphotic phenotype may place older men at risk for vertebral compression fractures due to the increased spinal load and impaired functional outcomes that are associated with greater kyphosis. While obesity did appear to have a protective effect on kyphosis in our cohort, intra-organ fat has been associated with poor health outcomes in older adults [44]. We are not advocating for obesity as a means to control excessive kyphosis, rather the need for more comprehensive assessment of health status among older adults with hyperkyphosis.

### Limitations

This study has a number of strengths including that the MrOS cohort includes community-dwelling older men residing in multiple geographic areas of the United States who were not preselected for hyperkyphosis. The kyphosis and the paraspinal muscle measurements were assessed at baseline with high reliability in a subgroup of study subjects. However, this study also has a number of limitations including possible misclassification of muscle measurements depending upon the exact location of the QCT scan slice. The paraspinal muscle group does increase in size closer its attachment sites, therefore our paraspinal muscle volume estimates measured at the L4-L5 intervertebral disc space may be underestimated, as would any association of paraspinal muscle volume and kyphosis found in this study. Another potential limitation is that we could not measure muscle volumes in the thoracic spine due to the CT technology used. However, our results suggest that lumbar paraspinal muscles serve as a proxy for muscle degeneration in the upper spine as well. We also used BMD measurements from the hip even though the muscle and kyphosis measurements were made in the spine. Hip BMD is often used in place of spine BMD because there is less artifact in the hip and the lumbar spine BMD from DXA can be overestimated because of extravertebral calcification (e.g. osteophytes). Hip BMD thus provides a more accurate measurement, particularly in elderly subjects. Moreover, when we replaced hip BMD with volumetric measurements of spine BMD with QCT, there was no appreciable difference in the results. The MrOS population may have degenerative spine conditions that could increase kyphosis and our regression analysis did not include adjustment for these degenerative changes. However, this is beyond the scope of this study. We chose to measure global Cobb angle using T4 and T12 endplates rather than other methods such as the centroid method that is less affected by endplate tilt [45] because the global method using the endplates of T4 and T12 is reliable and one of the most commonly used radiographic methods for measuring thoracic kyphosis. In future studies, we may consider using other methods that reflect changes in the middle of the curve. Finally, supine measurements of Cobb angle of kyphosis underestimate the degree of kyphosis compared to standing measurements, particularly among those with greater degree of kyphosis [27]. However, this would serve to underestimate any effects we found.

## Conclusions

In summary, we found that paraspinal muscle volume influenced kyphosis among older men with BMI < 30 kg/m^2^, and lower paraspinal muscle volume was associated with greater degree of thoracic kyphosis. Interestingly, prevalent vertebral fractures commonly thought to be the cause of excessive kyphosis, did not influence the association between paraspinal muscle volume, paraspinal IMAT and Cobb angle of kyphosis. Additional studies are now warranted that evaluate if low paraspinal muscle volume contributes to worsening of kyphosis in this population.

## Abbreviations

MrOS: Osteoporotic Fractures in Men; QCT: Quantitative computed tomography; BMD: Bone mineral density; SD: Standard deviation; IMAT: Intermuscular adipose tissue; BMI: Body mass index; kg/m2: Kilograms/meter^2^; HU: Hounsfield Unit; CI: Confidence interval; Health ABC: Health Aging and Body Composition Study; OHSU: Oregon Health and Science University; cm: Centimeter; ICC: Intraclass correlation coefficients.

## Competing interests

The authors declare that they have no competing interests.

## Authors’ contributions

WK conceived of the study, participated in its design and drafted the manuscript. DM carried out the statistical analysis. LM conceived of the study, participated in its design, drafted sections and edited the manuscript. NL participated in study design and edited the manuscript. DK conceived of the study, participated in study design and edited the manuscript. All authors read and approved the final manuscript.

## Pre-publication history

The pre-publication history for this paper can be accessed here:

http://www.biomedcentral.com/1471-2474/15/19/prepub

## References

[B1] Hirose D Posture of the trunk in the sagittal plane is associated with gait in community-dwelling elderly population Clin Biomech (Bristol, Avon) 2004 19 1 57 63 10.1016/j.clinbiomech.2003.08.005 14659931

[B2] Ryan SD Fried LP The impact of kyphosis on daily functioning J Am Geriatr Soc 1997 45 12 1479 1486 9400558 10.1111/j.1532-5415.1997.tb03199.x

[B3] Huang MH KW Cummings SR Kado DM Hyperkyphosis and decline in functional status in older community dwelling women: The Study of Osteoporotic Fractures in ASBMR 2010

[B4] Katzman WB Kyphosis and Decline in physical function over 15 years in older community-dwelling women: the study of osteoporotic fractures J Gerontol A Biol Sci Med Sci 2013 68 8 976 983 10.1093/gerona/glt009 23633167 PMC3712361

[B5] Kado DM Hyperkyphotic posture and poor physical functional ability in older community-dwelling men and women: the Rancho Bernardo study J Gerontol A Biol Sci Med Sci 2005 60 5 633 637 10.1093/gerona/60.5.633 15972617 PMC1360196

[B6] Takahashi T Trunk deformity is associated with a reduction in outdoor activites of daily living and life satisfaction in community-dwelling older people Osteoporos Int 2005 16 273 279 10.1007/s00198-004-1669-3 15235766

[B7] Briggs AM Thoracic kyphosis affects spinal loads and trunk muscle force Phys Ther 2007 87 5 595 607 10.2522/ptj.20060119 17472956

[B8] Bruno AG The effect of thoracic kyphosis and sagittal plane alignment on vertebral compressive loading J Bone Miner Res 2012 27 10 2144 2151 10.1002/jbmr.1658 22589006 PMC3431452

[B9] Bayliss M A conceptual and disease model framework for osteoporotic kyphosis Osteoporos Int 2013 24 9 2423 2432 10.1007/s00198-013-2317-6 23536254

[B10] Katzman W Association of Spinal Muscle Composition and Prevalence of Hyperkyphosis in Healthy Community-Dwelling Older Men and Women J Gerontol A Biol Sci Med Sci 2012 67 2 191 195 21878482 10.1093/gerona/glr160 PMC3297013

[B11] Kado D Factors associated with kyphosis progression in older women: 15 years experience in the Study of Osteoporotic Fractures J Bone Miner Res 2013 28 1 179 187 10.1002/jbmr.1728 22865329 PMC3693545

[B12] Kado DM Factors associated with kyphosis progression in older women: 15 years' experience in the study of osteoporotic fractures J Bone Miner Res 2013 28 1 179 187 10.1002/jbmr.1728 22865329 PMC3693545

[B13] Ropponen A Videman T Battie MC The reliability of paraspinal muscles composition measurements using routine spine MRI and their association with back function Man Ther 2008 13 4 349 356 10.1016/j.math.2007.03.004 17556006

[B14] Rossi A Quantification of intermuscular adipose tissue in the erector spinae muscle by MRI: agreement with histological evaluation Obesity (Silver Spring) 2010 18 12 2379 2384 10.1038/oby.2010.48 20300085 PMC5278643

[B15] Goodpaster BH Skeletal muscle attenuation determined by computed tomography is associated with skeletal muscle lipid content J Appl Physiol 2000 89 1 104 110 10904041 10.1152/jappl.2000.89.1.104

[B16] Kvist H Adipose tissue volume determination in males by computed tomography and 40 K Int J Obes 1988 12 3 249 266 3391740

[B17] Rossner S Adipose tissue determinations in cadavers–a comparison between cross-sectional planimetry and computed tomography Int J Obes 1990 14 10 893 902 2269582

[B18] Sjostrom L A computer-tomography based multicompartment body composition technique and anthropometric predictions of lean body mass, total and subcutaneous adipose tissue Int J Obes 1991 15 Suppl 2 19 30 1794934

[B19] Goodpaster BH The loss of skeletal muscle strength, mass, and quality in older adults: the health, aging and body composition study J Gerontol A Biol Sci Med Sci 2006 61 10 1059 1064 10.1093/gerona/61.10.1059 17077199

[B20] Goodpaster BH Attenuation of skeletal muscle and strength in the elderly: The health ABC study J Appl Physiol 2001 90 6 2157 2165 11356778 10.1152/jappl.2001.90.6.2157

[B21] Imamura K Human major psoas muscle and sacrospinalis muscle in relation to age: a study by computed tomography J Gerontol 1983 38 6 678 681 10.1093/geronj/38.6.678 6630901

[B22] Anderson DE Variations of CT-based trunk muscle attenuation by age, sex, and specific muscle J Gerontol A Biol Sci Med Sci 2013 68 3 317 323 10.1093/gerona/gls168 22904095 PMC3605905

[B23] Orwoll E Design and baseline characteristics of the osteoporotic fractures in men (MrOS) study–a large observational study of the determinants of fracture in older men Contemp Clin Trials 2005 26 5 569 585 10.1016/j.cct.2005.05.006 16084776

[B24] Blank JB Overview of recruitment for the osteoporotic fractures in men study (MrOS) Contemp Clin Trials 2005 26 5 557 568 10.1016/j.cct.2005.05.005 16085466

[B25] Miljkovic I Abdominal myosteatosis is independently associated to hyperinsulinemia and insulin resistance among older men without diabetes Obesity (Silver Spring) 2013 21 10 2118 2125 10.1002/oby.20346 23408772 PMC3661705

[B26] Marshall LM Dimensions and volumetric BMD of the proximal femur and their relation to age among older U.S. men J Bone Miner Res 2006 21 8 1197 1206 10.1359/jbmr.060506 16869717

[B27] Kado DM Christianson RN Palermo L Smith-Bindman R Cummings SR Greendale G Comparing a supine radiographic versus standing clinical measurement of kyphosis in older women: the fracture intervention trial Spine 2006 31 4 463 467 10.1097/01.brs.0000200131.01313.a9 16481959 PMC4964957

[B28] Schousboe JT Rosen HR Vokes TJ Cauley JA Cummings SR Nevitt MC Black DM Orwoll ES Kado DM Ensrud KE Prediction Models of Prevalent Radiographic Vertebral Fractures Among Older Men J Clin Densitom 2013 doi:10.1016/j.jocd.2013.09.020 10.1016/j.jocd.2013.09.020 PMC4035457 24289883

[B29] Genant HK Vertebral fracture assessment using a semiquantitative technique J Bone Miner Res 1993 8 9 1137 1148 8237484 10.1002/jbmr.5650080915

[B30] Hicks GE Trunk muscle composition as a predictor of reduced functional capacity in the health, aging and body composition study: the moderating role of back pain J Gerontol A Biol Sci Med Sci 2005 60 11 1420 1424 10.1093/gerona/60.11.1420 16339328

[B31] Hicks GE Cross-sectional associations between trunk muscle composition, back pain, and physical function in the health, aging and body composition study J Gerontol A Biol Sci Med Sci 2005 60 7 882 887 10.1093/gerona/60.7.882 16079212

[B32] Binder EF Effects of progressive resistance training on body composition in frail older adults: results of a randomized, controlled trial J Gerontol A Biol Sci Med Sci 2005 60 11 1425 1431 10.1093/gerona/60.11.1425 16339329

[B33] Fiatarone MA High-intensity strength training in nonagenarians. Effects on skeletal muscle JAMA 1990 263 22 3029 3034 10.1001/jama.1990.03440220053029 2342214

[B34] Frontera WR Strength conditioning in older men: skeletal muscle hypertrophy and improved function J Appl Physiol 1988 64 3 1038 1044 3366726 10.1152/jappl.1988.64.3.1038

[B35] Taaffe DR Alterations in muscle attenuation following detraining and retraining in resistance-trained older adults Gerontology 2009 55 2 217 223 10.1159/000182084 19060453 PMC2756799

[B36] Taaffe DR Comparative effects of high- and low-intensity resistance training on thigh muscle strength, fiber area, and tissue composition in elderly women Clin Physiol 1996 16 4 381 392 10.1111/j.1475-097X.1996.tb00727.x 8842574

[B37] Bennell KL Effects of an exercise and manual therapy program on physical impairments, function and quality-of-life in people with osteoporotic vertebral fracture: a randomised, single-blind controlled pilot trial BMC Musculoskelet Disord 2010 17 11 doi:10.1186/1471-2474-11-36 10.1186/1471-2474-11-36 PMC2830179 20163739

[B38] Bautmans I Rehabilitation using manual mobilization for thoracic kyphosis in elderly postmenopausal patients with osteoporosis J Rehabil Med 2010 42 2 129 135 10.2340/16501977-0486 20140408

[B39] Benedetti MG Effects of an adapted physical activity program in a group of elderly subjects with flexed posture: clinical and instrumental assessment J Neuroeng Rehabil 2008 5 32 10.1186/1743-0003-5-32 19032751 PMC2613395

[B40] Greendale GA Yoga Decreases Kyphosis in Senior Women and Men with Adult-Onset Hyperkyphosis: Results of a Randomized Controlled Trial J Am Geriatr Soc 2009 57 9 1569 1579 10.1111/j.1532-5415.2009.02391.x 19682114 PMC3700806

[B41] Itoi E Sinaki M Effect of back-strengthening exercise on posture in healthy women 49 to 65 years of age Mayo Clin Proc 1994 69 11 1054 1059 10.1016/S0025-6196(12)61372-X 7967758

[B42] Fon G Pitt M Thies A Thoracic kyphosis: Range in normal subjects AJR 1980 134 979 983 10.2214/ajr.134.5.979 6768276

[B43] Voutsinas SA MacEwen GD Sagittal profiles of the spine Clin Orthop 1986 210 235 242 3757369

[B44] Gastaldelli A Basta G Ectopic fat and cardiovascular disease: what is the link? Nutr Metab Cardiovasc Dis 2010 20 7 481 490 10.1016/j.numecd.2010.05.005 20659791

[B45] Briggs AM Radiographic measures of thoracic kyphosis in osteoporosis: Cobb and vertebral centroid angles Skeletal Radiol 2007 36 8 761 767 10.1007/s00256-007-0284-8 17437103

